# Heterogeneity of *Staphylococcus epidermidis* in prosthetic joint infections: time to reevaluate microbiological criteria?

**DOI:** 10.1007/s10096-021-04352-w

**Published:** 2021-10-02

**Authors:** Micael Widerström, Marc Stegger, Anders Johansson, Bharat Kumar Gurram, Anders Rhod Larsen, Lars Wallinder, Helen Edebro, Tor Monsen

**Affiliations:** 1 Department of Clinical Microbiology, Umeå University, SE-901 85 Umeå, Sweden; 2 Department of Bacteria, Parasites, and Fungi, Statens Serum Institut, Copenhagen, Denmark; 3 Department of Orthopaedics, University Hospital of Umeå, Umeå, Sweden

**Keywords:** *Staphylococcus epidermidis*, Genomics, Multidrug resistant, Prosthetic joint infection, Within-patient variation, Diagnosis, Polymicrobial

## Abstract

Prosthetic joint infection (PJI) is a feared and challenging to diagnose complication after arthroplasty, with *Staphylococcus epidermidis* as the major pathogen. One important criteria to define PJI is the detection of phenotypically indistinguishable microorganisms with identical antibiotic susceptibility pattern in at least two different samples. However, owing to phenotypical variation within genetic clones and clonal variation within a phenotype, the criteria may be ambiguous. We investigated the extent of diversity among coagulase-negative staphylococci (CoNS) in PJI and characterised *S. epidermidis* isolates from PJI samples, specifically multiple *S. epidermidis* isolates identified in individual PJI patients. We performed a retrospective cohort study on 62 consecutive patients with PJI caused by CoNS from two hospitals in Northern Sweden. In 16/62 (26%) PJIs, multiple *S. epidermidis* isolates were available for whole-genome analyses. Hospital-adapted multidrug-resistant genetic clones of *S. epidermidis* were identified in samples from 40/62 (65%) of the patients using a combination of pulsed-field gel electrophoresis and multilocus sequence typing. Whole-genome sequencing showed the presence of multiple sequence types (STs) in 7/16 (44%) PJIs where multiple *S. epidermidis* isolates were available. Within-patient phenotypical variation in the antibiotic susceptibility and/or whole-genome antibiotic resistance gene content was frequent (11/16, 69%) among isolates with the same ST. The results highlight the ambiguity of *S. epidermidis* phenotypic characterisation as a diagnostic method in PJI and call for larger systematic studies for determining the frequency of CoNS diversity in PJIs, the implications of such diversity for microbiological diagnostics, and the therapeutic outcomes in patients.

**Supplementary Information:**

The online version contains supplementary material available at 10.1007/s10096-021-04352-w.

## Introduction

Prosthetic joint replacement is one of the most important medical innovations of the twentieth century, and it has significantly improved the quality of life for millions of individuals worldwide by providing pain relief and restoring joint function, mobility, and independence [1, 2]. However, prosthetic joint infection (PJI) following joint replacement is a devastating complication associated with high medical costs and increased in-hospital mortality [3]. Both PJI diagnosis and treatment are challenging [4]. Previous studies have shown that *Staphylococcus aureus* and coagulase-negative staphylococci (CoNS), and in particular *Staphylococcus epidermidis*, are the pathogenic agents in most PJIs [5].

Currently, the international clinical guidelines for defining PJI diagnosis require two positive periprosthetic cultures with phenotypically ‘identical’ or ‘indistinguishable’ organisms, with the phenotype determined using common laboratory tests for genus and species identification and antibiograms [6–8]. Since phenotypic morphological variations [9], including the presence of small colony variants and different antibiograms, have been reported in monoclonal CoNS infections [10–12], the term ‘phenotypically identical organisms’ is ambiguous. Furthermore, the assessment of CoNS in clinical cultures is demanding as CoNS are a ubiquitous part of the human skin microbiota and often for *S. epidermidis* display high sub-species heterogeneity [13, 14], which makes distinction between contamination and true infection challenging. Here, we investigated the extent of diversity among CoNS in PJI and characterised in detail the *S. epidermidis* in these infections which revealed substantial within-patient diversity further highlighting the complexity and ambiguity in the current phenotypical assessment as the diagnostic criteria.

## Methods

### Study population

The patients in the study population were recruited from two hospitals in Northern Sweden: Umeå University Hospital and Östersund County Hospital. All patients were identified using the laboratory information systems and based on the presence of CoNS in more than two periprosthetic tissue biopsies retrieved during revision surgery in patients with clinically suspected PJI between December 2008 and June 2011. Diagnosis was based on the PJI diagnostic criteria of the Infectious Disease Society of America (IDSA, www.idsociety.org), ‘identical microorganisms isolated from two or more cultures’ [7], and classified according to the time point of occurrence after implantation: acute, within 1–3 months; delayed, 3 months to 1 year; late, after more than 1 year [6]. The medical records of the patients were reviewed for additional data on concomitant diseases, previous hospitalisation during the preceding year, intraoperative clinical findings by the surgeon, surgical treatment of PJI, and outcome at 2-year follow-up.

### Bacterial isolates

CoNS cultured from more than two perioperative tissue specimens (obtained during revision surgery of patients with clinically suspected PJI) were evaluated. Based on differences in colony morphology (i.e., size, consistency, luster, and colour), one to two isolates resembling CoNS were collected from each tissue culture for further investigation. The bacterial isolates were stored at − 70 °C in preservation media (Trypticase Soy Broth, BD Diagnostic Systems, Sparks, MD, USA) until further examination. In total, 131 CoNS isolates collected from 62 patients with PJIs were included for antimicrobial susceptibility testing and pulsed-field gel electrophoresis (PFGE). Only one isolate was available for PFGE and MLST in 40/62 (65%) patients with PJIs. Multiple CoNS isolates were available from 22 PJIs; 18 caused by *S. epidermidis*, two *Staphylococcus* *capiti*s, one *Staphylococcus* *caprae*, and one *Staphylococcus hominis*. We were not able to recultivate multiple isolates of *S. epidermidis* for WGS in two PJIs*.* Hence, multiple *S. epidermidis* (*n* = 69) isolates collected from 16 patients were available for whole-genome sequencing (WGS) (Supplementary Figs. S1 and S2).

### Identification

Species-level identification was performed using matrix-assisted laser resorption/ionisation time-of-flight mass spectrometry (MALDI-TOF MS) with a Microflex LT (Bruker Daltonik GmbH, Bremen, Germany) and MALDI Biotyper software v3.1 DB7311 (Bruker Daltonik), according to the manufacturer’s instructions. A score > 2.0 was required for species identification [15].

### Antimicrobial susceptibility testing

Antimicrobial susceptibility was tested using disc diffusion according to the recommendations of the European Committee on Antimicrobial Susceptibility Testing (EUCAST, http://www.eucast.org) with the following eight antimicrobials: cefoxitin, clindamycin, erythromycin, fusidic acid, gentamicin, norfloxacin, rifampicin, and trimethoprim/sulfamethoxazole. The clinical breakpoints were the same as those specified in the EUCAST recommendation (v10.0). Vancomycin heteroresistance was not tested. Multidrug resistance (MDR) was defined as resistance towards antimicrobials from more than three classes.

### PFGE and multilocus sequence typing (MLST)

PFGE and MLST were performed as previously described [16]. In short, DNA was prepared from 3 mL *S. epidermidis* overnight cultures in Todd Hewitt broth (Difco Laboratories). The DNA was digested using *Sma*I (Thermo Fisher Scientific, Waltham, MA, USA), and the DNA fragments were separated by PFGE in a GenePath apparatus (Bio-Rad Laboratories) using Program 14, for 19.7 h according to the manufacturer’s instructions (Bio-Rad). Gels were stained in 1 mg/L ethidium bromide, destained, and photographed under UV illumination. Genetic similarity between isolates was calculated using GelCompar II 4.0 (Applied Maths) using the Dice coefficient, and the unweighted pair group method with arithmetic mean (UPGMA) with 1.3% tolerance and 0.8% optimization settings. *S. aureus* NCTC 8325 was included as a reference in every sixth to seventh lane to allow normalisation of the electrophoretic pattern. Band sizes below 36 kb were not analysed. PFGE types were visually identified according to established criteria; isolates with more than three-band variation in the PFGE pattern were defined as genetically unrelated. This corresponded to a similarity coefficient of 90% using the cluster analysis.

PFGE was performed using all isolates (*n* = 131). All unique *S. epidermidis* PFGE types that included at least three isolates were further analysed using MLST (*n* = 102) according to Thomas et al. [17]. The 7 housekeeping genes were amplified by PCR, and both strands of all amplicons were sequenced with an Applied Biosystems 3730xl DNA Analyser by using BigDye Terminator v3.1 cycle sequencing kit. Sequence types (STs) were assigned using the *S. epidermidis* MLST database (https://pubmlst.org/sepidermidis/).

### Genome sequencing and analyses

WGS was performed on the 69 *S. epidermidis* isolates collected from 16 patients with PJI using Illumina MiSeq and the 300-cycle MiSeq Reagent Kit v3 to generate paired-end 150-bp reads according to the manufacturer’s instructions. DNA purification was performed using the Qiagen Blood and Tissue kit. The sequencing data were subjected to quality control using bifrost (https://github.com/ssi-dk/bifrost) to ensure adequate sequencing quality of all isolates and assembled using SPAdes v3.9.0 [18]. After the duplicated regions in the reference were eliminated using NUCmer, the raw reads were aligned against the *S. epidermidis* ATCC 12,228 reference chromosome (GenBank accession ID CP0222479) for detecting single nucleotide polymorphisms (SNPs) using NASP v1.0.0 [19]. NASP was also used to detect intraspecies contamination. All positions with < tenfold coverage or cases where the variant was present in < 90% of the base calls were excluded using GATK [20]. The SNPs identified in the core genome were used to infer phylogenetic relationships using PhyML v3 [21] with Smart Model Selection [22]. The resistance mechanisms were determined as previously described [23].

### Statistics

Statistical analyses were performed using the SPSS v24 (SPSS Inc., Chicago, IL, USA) software package. Fisher’s exact test was used to test association in all two-way tables. A value with *p* < 0.05 was considered significant.

### Research ethics

The study was approved by the Research Ethics Committee (No 2012–477-31 M) of the Faculty of Medicine, Umeå University, Sweden. All study participants provided written informed consent prior to study participation.

## Results

### Clinical characteristics

The clinical characteristics of 62 consecutive patients (34 men and 28 women; median age 68.6 years) requiring revision or resection arthroplasties for CoNS-related PJIs are presented in Table 1.

**Table 1:** Intra-lineage pairwise single-nucleotide polymorphism Demographic and clinical characteristics of 62 consecutive patients with prosthetic joint infections caused by coagulase-negative staphylococci

Characteristics	Total (%)
Median age (IQR), year	68.6 (60.5 − 77.5)
No. of female patients	28 (45)
Arthroplasty	
Hip	38 (61)
Knee	21 (34)
Other: elbow (*n* = 1), shoulder (*n* = 2)	3 (5)
Reason for primary arthroplasty	
Osteoarthritis	36 (60)
Fracture or trauma	15 (32)
Rheumatoid arthritis	11 (8)
IDSA PJI criteria	
Identical organism identified with two separate cultures	62 (100)
Presence of sinus tract	9 (15)
Visible purulence at implant site	52 (84)
Surgical procedure	62 (100)
Debridement and implant retention	21 (34)
Revision (one-stage exchange)	6 (10)
Resection (two-stage exchange or resection)	35 (56)
Type of revision	
Primary	35 (56)
Secondary	27 (44)
Timing from prosthesis implanted to surgery (month)	
Early onset (< 3)	37 (60)
Delayed onset (3 to 12)	11 (18)
Late onset (> 12)	14 (22)
Hospitalisation in previous year	
Yes	36 (58)
No	26 (42)
Failure of 2-year follow-up	
Yes	15 (24)
No	47 (76)

*IQR* inter-quartile range, *PJI* prosthetic joint infection, *IDSA* Infectious Disease Society of America

### Microbiological findings and genetic analyses

**Fig. 1:**
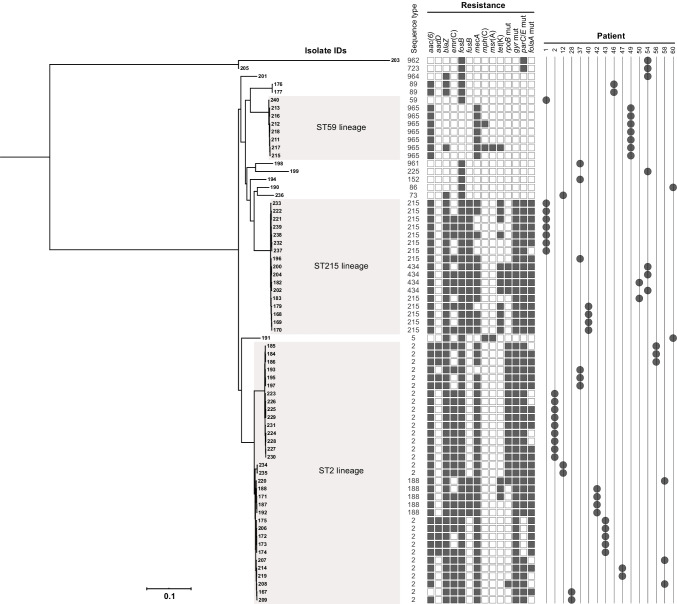
Midpoint-rooted maximum-likelihood phylogeny of 69 *Staphylococcus* *epidermidis* prosthetic joint infection isolates from 16 patients. Information on sequence type, genes associated with antimicrobial resistance is presented along with the patient number. The scale bar indicates the substitutions per site. The black blocks indicate the presence of genes mediating antibiotic resistance or previously associated with virulence

In total, 131 CoNS isolates as determined using MALDI-TOF MS were available from 62 patients with PJI, with *S. epidermidis* (*n* = 107; 85%), *Staphylococcus capitis* (*n* = 11; 8%), and *Staphylococcus hominis* (*n* = 8; 4%) as the most frequent species (Table 2, Supplementary Fig. S1). PFGE analysis of all 131 isolates and subsequent MLST on selected *S. epidermidis* PFGE types revealed two major clusters corresponding to ST215 and ST2 (Supplementary Fig. S2). In addition, two single locus variants (SLVs) of ST215 (ST434) and ST2 (ST188) were identified, and the major clusters, including the SLVs, were denoted as the ST215 and ST2 lineages, respectively (Fig. 1). Among *S. epidermidis* isolates, the antimicrobial resistance rates were as follows: cefoxitin (80%), gentamicin (90%), norfloxacin (79%), trimethoprim-sulfamethoxazole (75%), clindamycin (63%), fusidic acid (42%), and rifampicin 33% (Supplementary Table S2). No resistance to linezolid was detected. Significant differences in antimicrobial susceptibility were identified when the two major genetic clusters were compared. All 32 isolates of the ST215 lineage exhibited fusidic acid resistance compared to 21% in the ST2 lineage (*p* < 0.0001). In contrast, rifampicin resistance was significantly more common among isolates in the ST2 lineage (27/47, 57%) than in the ST215 lineage (5/32, 16%) (*p* = 0.0002).

**Table 2:** Microbiological characteristics of isolates from 62 consecutive patients with prosthetic joint infections

Microbiology	Monomicrobial *n* = 43 (69%)	Polymicrobial *n* = 19 (31%)	Total *n* = 62
	*n*	*n*	*n*
*Staphylococcus epidermidis*	33	19	52
*S. capitis*	5	0	5
*S. hominis*	2	3	5
*S. lugdunensis*	1	1	2
*S. caprae*	1	0	1
*S. warneri*	1	0	1
*S. haemolyticus*		1	1
*Enterococcus faecalis*^*a*^		7	7
*Escherichia coli*^*a*^		3	3
*S. aureus*^*a*^		2	2
*S. dysgalactiae* (GGS)		1	1
*Cutibacterium acnes*		1	1
*Peptostreptococcus*		1	1
*Enterobacter*		1	1

^*a*^In two cases, three pathogens were identified in multiple tissue biopsies: *S. epidermidis*, *S. aureus*, and *Enterococcus faecalis* (patient ID 10) and *S. epidermidis*, *Escherichia* *coli*, and *Enterococcus faecalis* (patient ID 47)

### Phenotypic diversity of CoNS in PJI

The majority (43/62; 69%) of the PJIs were monomicrobial, with *S. epidermidis* detected in most PJIs (*n* = 33), followed by *S. capitis* (*n* = 5) and *S. hominis* (*n* = 2) (Table 2, Supplementary Table S3). *S. epidermidis* was also detected in all 19 polymicrobial PJIs, most frequently in combination with *Enterococcus faecalis* (*n* = 7), *Escherichia coli* (*n* = 3), or *S. hominis* (*n* = 2), and with similar frequencies in hip and knee revision arthroplasties with 11/37 (30%) and 6/20 (33%), respectively.

The presence of a sinus tract with communication to the joint in PJI was reported in only 2/19 (11%) polymicrobial *S. epidermidis* PJIs.

### Whole-genome analyses of S. epidermidis in PJIs

**Fig. 2:**
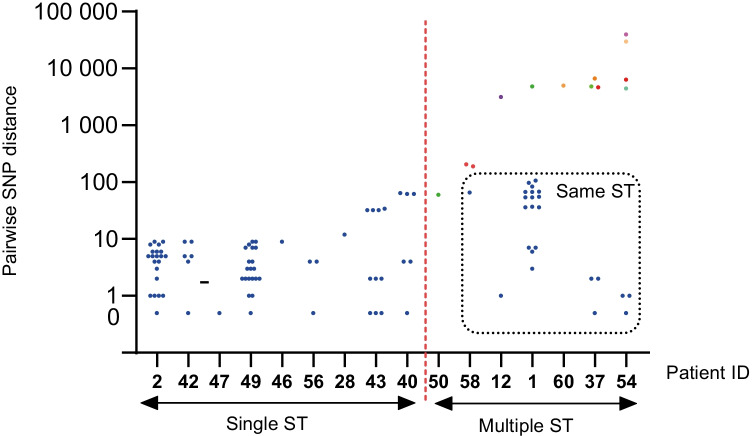
Pairwise within-patient single-nucleotide polymorphism distances among 16 patients with prosthetic joint infection, with more than two *Staphylococcus epidermidis* isolates available for analysis. Different colours indicate different sequence types

**Fig. 3:**
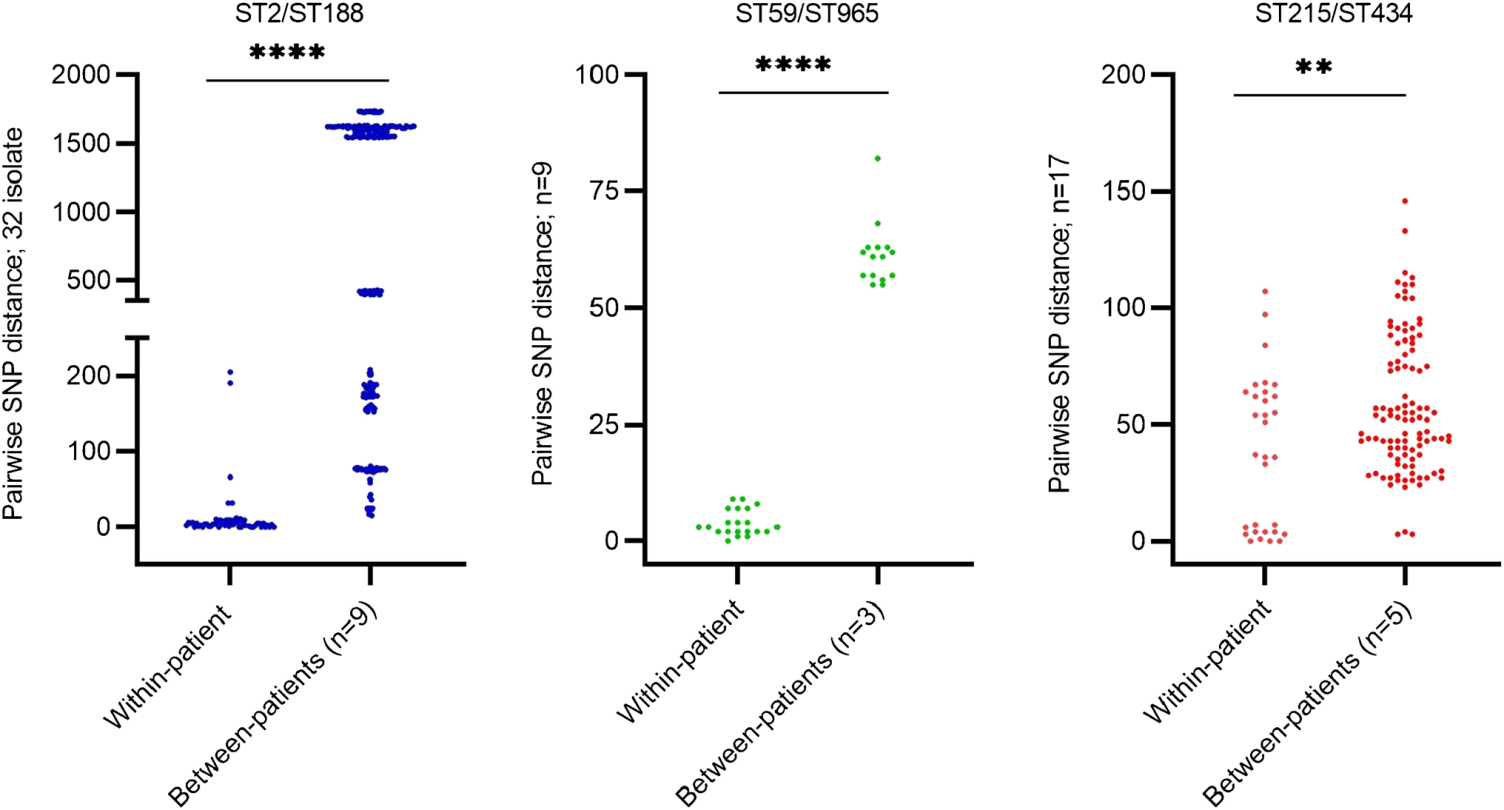
Pairwise single-nucleotide polymorphism (SNP) within-patient and between-patients SNP distances among prosthetic joint infection *Staphylococcus* *epidermidis* isolates from ST2/ST188, ST59/ST965, and ST215/ST434. ****p < 0.0001, **p = 0.002

In 16 of the 62 cases of PJI, 9 of which were monomicrobial infections, multiple *S. epidermidis* isolates (*n* = 69) from different samples collected from the same patient were available for WGS (Supplementary Figs. S1 and S3, Table 3). Between two to nine isolates were available per patient, and genomic analysis of these isolates helped identify three major lineages: ST59/ST965 in two PJIs, ST215 in five PJIs, and ST2 in nine PJIs (Fig. 1). Nine of the sixteen (56%) patients were infected by bacteria from a single *S. epidermidis* lineage, whereas seven (44%) patients were infected by bacteria from two to five different *S. epidermidis* lineages (Fig. 2). Based on a 73% (1.83 Mb) core genome conservation across all the sequenced *S. epidermidis* PJI isolates, we found that the within-patient genetic diversity among isolates from individual STs ranged from 10 to 107 SNPs, whereas among isolates from PJIs caused by bacteria from multiple STs, 100 to 39,618 SNPs were detected (Fig. 3, Supplementary Table S1).

**Table 3:** Within-patient phenotypic and genotypic polymorphisms among 16 patients with prosthetic joint infection caused by *Staphylococcus* *epidermidis* with more than two isolates, as analysed using whole-genome sequencing

Patient ID	Type of implant	Classification^a^	No. of specimens included	No. of *Staphylococcus epidermidis* isolates	No. of different antibiograms	Sequence type(s)	Polymicrobial	Additional species
2	Hip	Early	7	9	1	2	No	
12	Hip	Early	3	3	2	2, 73	Yes	*S. hominis*
28	Hip	Early	2	2	2	2	No	
46	Hip	Early	2	2	1	89	No	
47	Hip	Delayed	2	2	1	2	Yes	*Enterococcus faecalis: Escherichia coli*
50	Hip	Delayed	2	2	2	215, 434	Yes	*E. faecalis*
56	Hip	Delayed	2	3	2	2	No	
1	Hip	Late	4	8	5	59, 215	Yes	*S. hominis*
40	Hip	Late	4	4	2	215	Yes	*S. haemolyticus*
43	Knee	Early	5	5	2	2	No	
54	Knee	Early	6	7	3	225, 434, 723, 962, 964	Yes	*S. hominis*
58	Knee	Early	3	3	3	2, 188	No	
60	Knee	Early	2	2	2	5, 86	Yes	*S. lugdunensis*
42	Knee	Delayed	4	4	2	188	No	
49	Knee	Late	7	7	3	965	No	
37	Shoulder	Late	5	6	4	2, 152, 215, 961	No	

^a^Early =  < 3 months, delayed = 3–12 months, late =  > 12 months

### Resistance determinants in S. epidermidis lineages

The presence of phenotypic antimicrobial resistance and antimicrobial resistance genes identified using WGS was highly concordant (Supplementary Table S4). Our analyses showed that some antibiotic resistance genes were also associated with the STs (Fig. 1, Supplementary Table S1): *fusB* acid *and tet*(K), conferring resistance to fusidic acid and tetracycline respectively, were detected only in ST215/ST434, ST188, and ST59. Likewise, mutations in *gyr*(A), conferring resistance to fluoroquinolones, were detected in isolates belonging to ST2, ST22, and ST215, whereas *rpoB* mutations conferring rifampicin resistance were identified in both ST2 lineages and in ST434 isolates, but not in any of the ST215 isolates (Fig. 1, Table S1).

### Within-patient variations in phenotypic and genotypic resistance

When we compared multiple *S. epidermidis* isolates collected from each patient with PJI, variations in antibiograms were identified in 13 of the 16 (81%) cases (Table 4). Differences in susceptibility to one to five antimicrobials were observed (Fig. 1, Table 3), with colony polymorphism among isolates observed in samples collected from all patients (data not shown). The variation in detected antibiotic resistance genes was also apparent when multiple isolates of the same ST collected from one patient were compared (Fig. 1, Supplementary Table S1); in patient 1, six out of seven ST215 isolates varied in genetic content with respect to *mecA*, *tet*(K), *ermC*, and mutations in *folA*.

**Table 4:** Within-patient polymorphism in phenotypic antimicrobial susceptibility patterns among 16 patients with prosthetic joint infections with more than two isolates of *Staphylococcus epidermidis*

Patient ID	ST	Isolates (*n*)	Antimicrobial							
			FUS	FOX	GEN	CLI	ERY	NOR	RIF	SXT
1	59	1	R	S	S	S	S	S	S	S
	215	2	R	S	R	R	R	R	S	R
	215	1	R	R	R	S	S	R	S	R
	215	2	R	S	R	S	S	R	S	R
	215	2	R	R	R	R	R	R	S	R
2	2	9	S	R	R	R	R	R	R	R
12	2	2	S	R	R	R	R	R	R	R
	73	1	S	S	S	S	S	S	S	S
28	2	1	S	R	S	R	R	R	S	R
	2	1	R	R	R	R	R	R	S	R
37	2	1	S	S	R	R	R	R	R	R
	2	2	S	R	R	S	S	R	R	R
	152	1	S	S	S	S	S	S	S	S
	215	1	R	R	R	R	R	R	S	R
	961	1	S	S	S	S	S	S	S	S
40	215	3	R	R	R	S	S	R	S	R
	215	1	R	S	R	R	R	R	S	R
42	188	3	R	R	R	R	R	R	S	R
	188	1	R	R	R	S	S	R	S	R
43	2	4	S	R	R	R	R	R	S	R
	2	1	S	R	R	S	S	R	S	R
46	89	2	S	S	R	S	S	S	S	S
47	2	2	S	R	R	R	R	R	S	R
49	965	5	S	R	R	S	S	S	S	S
	965	1	R	R	R	S	R	S	S	S
	965	1	S	R	R	S	R	S	S	S
50	215	1	R	R	R	R	R	R	S	R
	434	1	R	R	R	R	R	R	R	R
54	225	1	S	S	S	S	S	S	S	S
	434	3	R	R	R	R	R	R	R	R
	723	1	S	S	R	S	S	S	S	S
	962	1	S	S	S	S	S	S	S	S
	964	1	S	S	S	S	S	S	S	S
56	2	2	S	R	R	S	S	R	R	R
	2	1	S	R	R	R	R	R	R	R
58	2	1	S	R	R	R	R	R	S	R
	2	1	S	R	R	R	R	R	R	R
	188	1	R	R	R	R	R	R	R	R
60	5	1	S	S	R	S	R	S	S	S
	86	1	S	S	R	S	S	S	R	S

*GEN* gentamicin, *CLI* clindamycin, *ERY* erythromycin, *FUS* fusidic acid, *FOX* cefoxitin, *NOR* norfloxacin, *RIF* rifampicin, *SXT* trimethoprim-sulfamethoxazole, *R* resistant, *S* susceptible

## Discussion

Here, we investigated the diversity among CoNS in PJI and characterised in detail the *S. epidermidis* isolates from these infections. We found considerable within-patient diversity in *S. epidermidis* isolates, with variations in phenotypic and genotypic resistance observed in the majority (13/16; 81%) of cases, and also between isolates with the same ST. Additionally, while we considered the inherent difficulty of ruling out the possibility that a single *S. epidermidis* isolate represents contamination, *S. epidermidis* isolates belonging to different STs were detected in several PJIs (7/16; 44%). These findings further add to the complexity in assessing whether *S. epidermidis* identified in multiple cultures in potential PJI cases represent phenotypically identical organisms in two positive periprosthetic cultures. Hence, with the present guidelines, there is a risk that PJI pathogens are incorrectly dismissed as contaminants, which hinders the appropriate microbial diagnosis and treatment.

To date, limited data has been published on the within-patient genetic diversity of CoNS isolates in PJI. Here, we showed that while only a single ST was detected in the majority (9/16, 56%) of the PJIs, polyclonality was detected in 44% (7/16) of all PJIs with between two to five different STs. Importantly, when at least three *S. epidermidis* isolates from different tissue samples were characterised in each PJI, the extent of the identified within-patient diversity among *S. epidermidis* isolates increased. Within-patient variation in antibiograms was observed comparing *S. epidermidis* isolates in almost all patients (10/11, 91%) and different STs were identified in 5/11 (45%). Obviously, among PJIs in which only two *S. epidermidis* isolates were available for characterisation, polyclonal infection was detected less frequently (2/5 patients; Table 3). These results are consistent with those of a recent German study in which paired isolates from 55 cases of orthopaedic device-related infection were analysed, and 6/55 (11%) cases assessed as polyclonal [24]. Therefore, increasing the number of *S. epidermidis* isolates for characterisation, and preferably obtaining isolates from different tissue specimens, is important for determining isolate diversity and reduces the risk of incorrect dismissal of isolates as contaminants, and improves the basis for decisions on antibiotic therapy and accurate identification of a relapse or reinfection.

In agreement with previous data, isolates from HA-MDRSE lineages were the cause of most *S. epidermidis* PJIs over a period of more than 2 years in the two hospitals in Northern Sweden [23, 25]. The low pairwise diversity in the ST215 lineage observed in two PJI cases for which isolates were collected more than 1 year apart in the same hospital (2 SNPs), indicates that the ST215 lineage is persistent in the hospital setting in Sweden. In contrast, limited hospital-adapted transmission of genetic lineages has been reported in *S. aureus* PJIs [26]. The findings of this study are in alignment with a previously described scenario of the global dissemination of multidrug-resistant lineages of *S. epidermidis* [27, 28]. The likelihood of hospital-adapted transmission was further corroborated by a recent large study on *S. epidermidis* PJI in Sweden [23]. The adaptation of ST2 and ST215 lineages to the hospital environment includes common genomic traits (*IS*256) and high prevalence of antimicrobial resistance genes even though some lineage-dependent differences are evident, i.e., the ST215 lineage lacks the intercellular adhesion gene A (*icaA*) gene [23, 27]. The primary source of HA-MDRSE lineages and the routes of transmission are uncertain. Recent data suggest that current perioperative PJI prevention regimens select MDRSE either from the patient’s normal flora or by facilitating acquisition from the hospital environment [23].

Polymicrobial infections, including those caused by *S.* *epidermidis*, were common among the PJIs investigated in this study, and consistent with previous data, *Enterococcus* *faecalis* was the most frequent companion microbe [29]. In most cases, both *S. epidermidis* and *E. faecalis* were detected in most tissue specimens from each patient, which reduced the chances of contamination; however, the possibility cannot be completely excluded.

The results presented here have practical and clinical implications. The within-patient diversity of *S. epidermidis* infections suggests that the clinical microbiology assessment of a PJI requires re-evaluation [30]. We believe characterising more than two isolates phenotypically and genotypically will improve assessment regardless of whether the PJI microbiological diagnostic criteria are met. In-depth analysis of more than two isolates will also provide additional information for selecting the appropriate targeted antibiotic therapy and help distinguish between a relapse and reinfection. The present clinical microbiology method for genetic heterogeneity assessment is laborious and expensive; however, advances reported in recent studies may change that in the near future. For example, new culture-independent methods that can be applied in clinical laboratories can facilitate the rapid assessment of clonality and population structure of *S. epidermidis* communities in PJI [31]. Another suitable approach is the culturing of several specimens followed by sequencing of multiple microbial isolates during routine PJI microbial diagnostics. Given the high cost of PJI treatment, it is practical to implement routine PJI diagnostics using small-scale rapid sequencing technologies with a turn-around time, including bioinformatics, of 1–2 days [32].

There were a few limitations to this study. First, the study had a retrospective cohort design. However, this may not affect the investigation of *S. epidermidis* populations causing PJIs, as it was shown that the *S. epidermidis* population structure in central Sweden has remained fairly stable over the last 10 years [23]. Second, the collection of more isolates per patient would strengthen our findings. Multiple *S. epidermidis* isolates for WGS analysis could only be collected from a few patients with PJI. That said, the microbiological findings of heterogeneity indicate that the present-day criteria for PJI diagnosis is sub-optimal. Third, and most importantly, some of the isolates detected and characterised were potential contaminants and were not truly invasive; however, all consecutive patients with PJI who met the IDSA criteria were included. Further, we used fresh sets of skin incision and subcutaneous incision instruments and a new set of sterile instruments for each tissue specimen to reduce the risk of contamination.

In conclusion, the within-patient genetic diversity in *S. epidermidis* isolates was substantial, with variation in both antibiotic susceptibility and antibiotic resistance genes. The findings highlight the complexity and ambiguity in the phenotypical assessment of CoNS isolates from periprosthetic tissue cultures as diagnostic criteria for PJI. Larger systematic studies are needed to determine the implications of these findings for microbiological diagnosis and the clinical significance of these results for therapeutic outcomes.

## Supplementary Information

Below is the link to the electronic supplementary material.

Supplementary file1 (XLSX 30 KB)

Supplementary file2 (PDF 666 KB)

## Data Availability

The whole-genome sequence data generated in this study have been submitted to the European Nucleotide Archive under BioProject ID PRJEB44086.
