# Supplementary material for: Heterogeneity of Staphylococcus epidermidis in prosthetic joint infections: time to reevaluate microbiological criteria?
Source: Eur J Clin Microbiol Infect Dis. 2021 Oct 2;41(1):87–97. doi: 10.1007/s10096-021-04352-w (PMC8732909; doi:10.1007/s10096-021-04352-w)
Supplement: Supplementary file 2 — Supplementary file2 (PDF 666 KB) [file 10096_2021_4352_MOESM2_ESM.pdf]

Fig S1. Flowchart depicting the 62 CoNS PJI patients included in the study and the 16 *S. epidermidis* prosthetic joint infections with multiple isolates analyzed by WGS

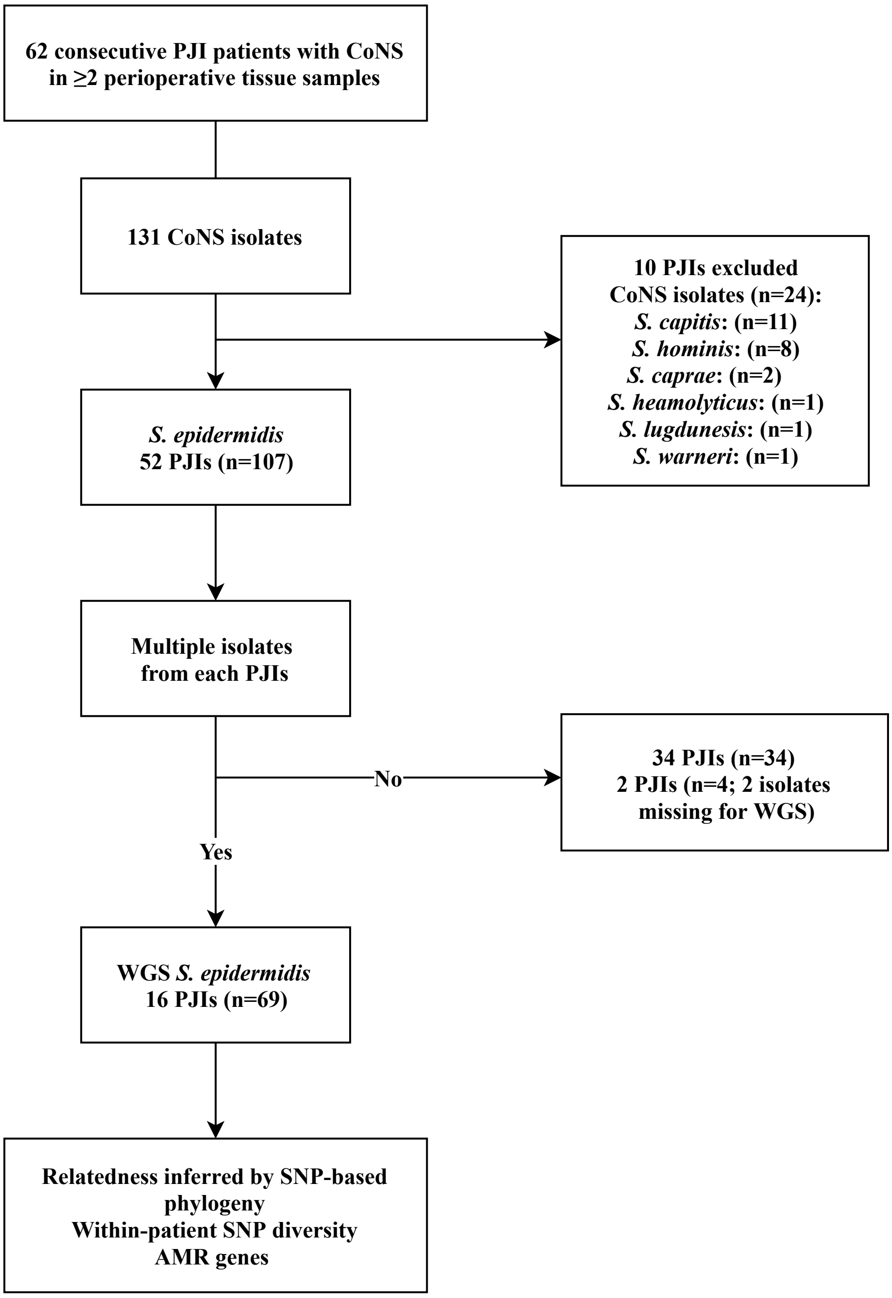

Fig. S2. Dendrogram cluster analysis of the genetic similarity of 131 CoNS isolates using pulsed-field gel electrophoresis (PFGE). The horizontal upper bar indicates genetic similarity (per cent). The dotted lines in the centre of the diagram represent digitalized transformation of the PFGE DNA pattern. The columns to the right present the following: patient ID, PFGE type or non-*S. epidermidis* species and sequence type (ST).

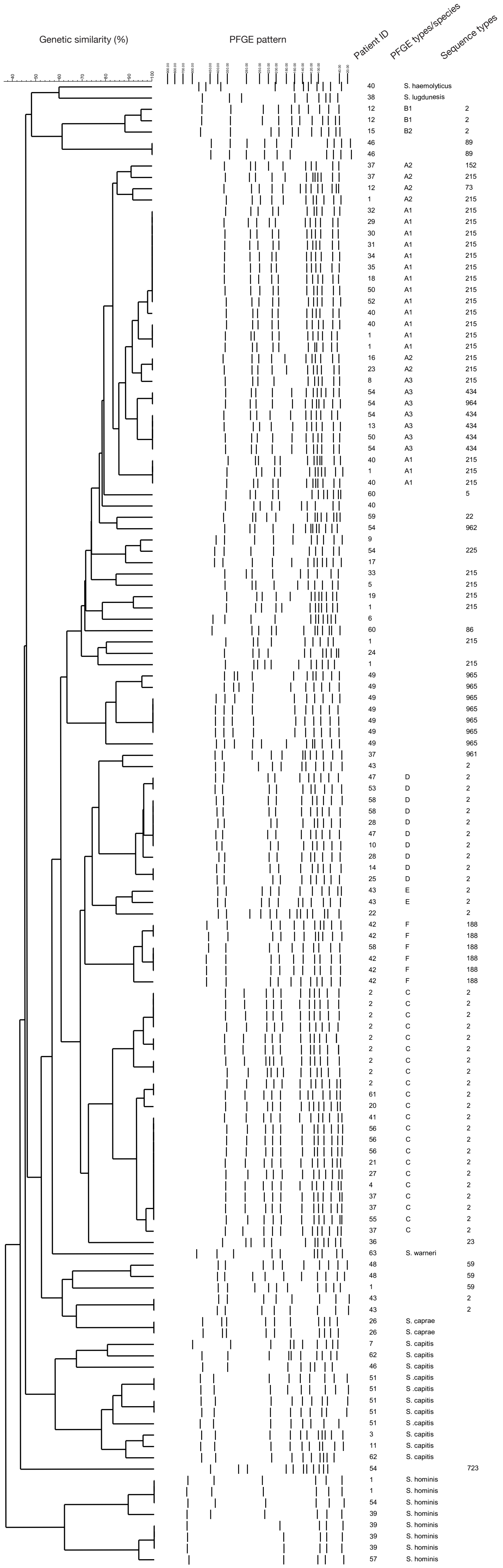
Genetic similarity (%)
PFGE pattern
Patient ID
PFGE types/species
Sequence types
40
50
60
70
80
90
100
40 S. haemolyticus
38 S. lugdunesis
12 B1 2
12 B1 2
15 B2 2
46 89
46 89
37 A2 152
37 A2 215
12 A2 73
1 A2 215
32 A1 215
29 A1 215
30 A1 215
31 A1 215
34 A1 215
35 A1 215
18 A1 215
50 A1 215
52 A1 215
40 A1 215
40 A1 215
1 A1 215
1 A1 215
16 A2 215
23 A2 215
8 A3 215
54 A3 434
54 A3 964
54 A3 434
13 A3 434
50 A3 434
54 A3 434
40 A1 215
1 A1 215
40 A1 215
60 5
40
59 22
54 962
9
54 225
17
33 215
5 215
19 215
1 215
6
60 86
1 215
24
1 215
49 965
49 965
49 965
49 965
49 965
49 965
49 965
37 961
43 2
47 D 2
53 D 2
58 D 2
58 D 2
28 D 2
47 D 2
10 D 2
28 D 2
14 D 2
25 D 2
43 E 2
43 E 2
22 2
42 F 188
42 F 188
58 F 188
42 F 188
42 F 188
42 F 188
2 C 2
2 C 2
2 C 2
2 C 2
2 C 2
2 C 2
2 C 2
2 C 2
2 C 2
61 C 2
20 C 2
41 C 2
56 C 2
56 C 2
56 C 2
21 C 2
27 C 2
4 C 2
37 C 2
37 C 2
55 C 2
37 C 2
36 23
63 S. warneri
48 59
48 59
1 59
43 2
43 2
26 S. caprae
26 S. caprae
7 S. capitis
62 S. capitis
46 S. capitis
51 S .capitis
51 S .capitis
51 S. capitis
51 S. capitis
51 S .capitis
3 S. capitis
11 S. capitis
62 S. capitis
54 723
1 S. hominis
1 S. hominis
54 S. hominis
39 S. hominis
39 S. hominis
39 S. hominis
39 S. hominis
57 S. hominis

Fig S3. Intra-lineage pairwise SNP diversity among *S. epidermidis* STs within each patient with PJI related to time since primary surgery

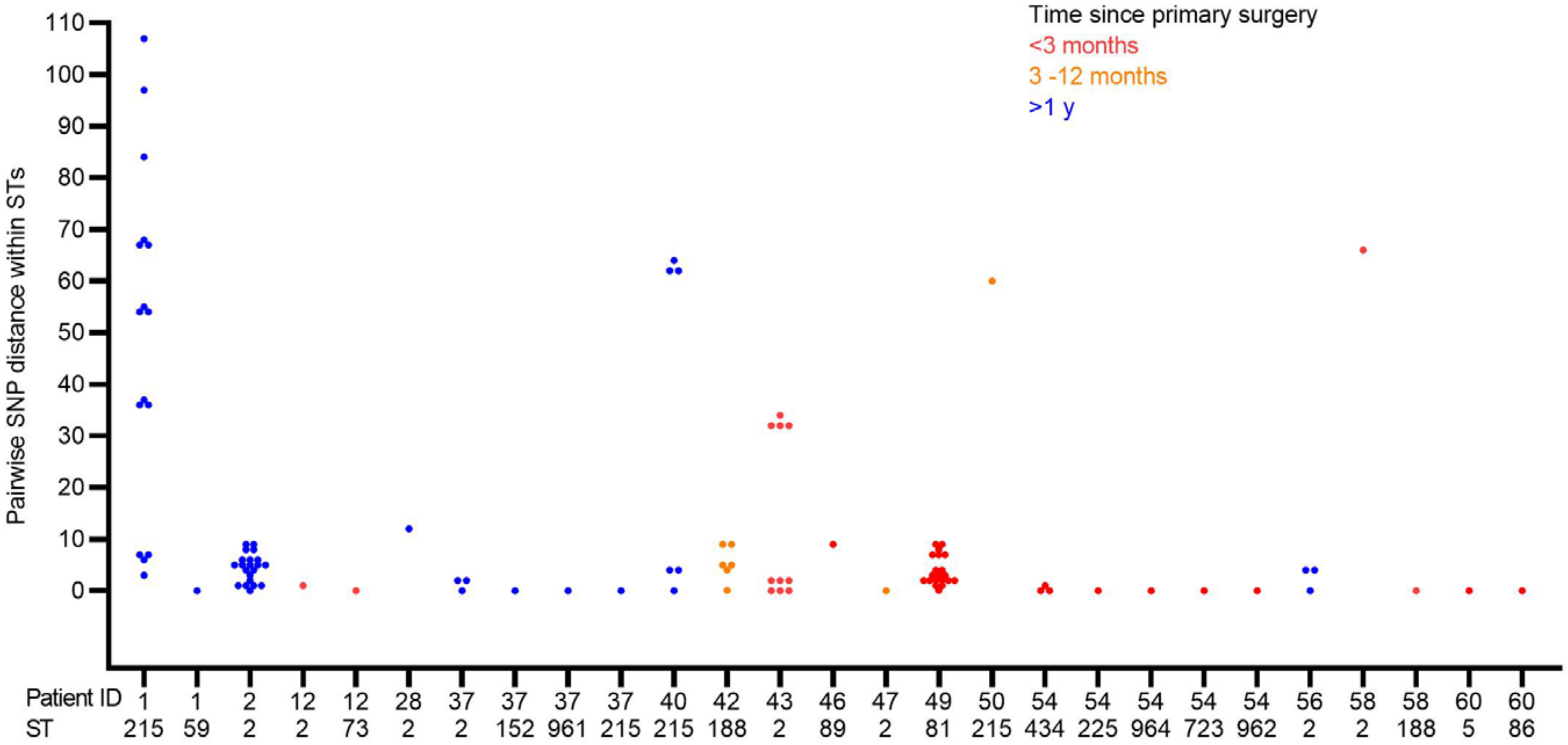

Table S2. Antimicrobial susceptibility pattern among 107 consecutive *S. epidermidis* isolates identified in 62 patients with prosthetic joint infections identified in two Swedish hospitals between 2009 and 2011

		Antimicrobial phenotypic resistance (%)								
	n	GEN	CLI	ERY	FUS	FOX	NOR	RIF	SXT	MDR
S. epidermidis	107	96 (90)	67 (63)	72 (67)	45 (42)	86 (80)	85 (79)	35 (33)	80 (75)	87 (81)
ST2/ST188	47	45 (96)	38 (81)	39 (83)	10 (21)	45 (96)	47 (100)	27 (57)	46 (98)	47 (100)
ST215/ST434	32	32 (100)	24 (75)	24 (75)	32 (100)	26 (81)	32 (100)	5 (16)	31 (97)	32 (100)
Non ST2/ST215[a]	28	19 (68)	5 (18)	9 (32)	3 (11)	15 (54)	6 (21)	2 (7)	4 (14)	8 (29)

GEN= gentamicin; CLI=clindamycin; ERY=erythromycin; FUS=fusidic acid; FOX=cefoxitin; NOR=norfloxacin; RIF=rifampicin; SXT=trimethoprim-sulfamethoxazole. MDR=multidrug-resistant, defined as resistance towards ≥3 antimicrobial categories

[a]ST5, ST22, ST23, ST86, ST89, ST152, ST225, ST723, ST961, ST962, ST964, ST965

Table S3. Distribution of *S. epidermidis* lineages in 52 consecutive patients with monomicrobial or polymicrobial prosthetic joint infections

Lineage	Monomicrobial (%)	Polymicrobial (%)	Total (%)
ST215[c]	9 (27)	9 (47)	18 (35)
ST2[c]	16 (49)	6 (32)	22 (42)
Non-ST215/ST2[d]	8 (24)	4 (21)	12 (23)
Total	33	19	52

[c]In patient 37, several STs were detected: ST2 (*n*=3), ST215 (*n*=1), ST152 (*n*=1) and ST961 (*n*=1). Patient 37 is included among ST2/188 cases due to several isolates of ST2

[d]ST5, ST22, ST23, ST86, ST89, ST152, ST225, ST723, ST961, ST962, ST964, ST965

Table S4. Agreement between genomic presence/absence of a gene or a gene mutation encoding antimicrobial resistance and a phenotypical antimicrobial resistance/susceptibility trait

Antimicrobial resistance				Agent	Agreement (%)
Gene	*n*	phenotypic			
		R	S		
mecA +	51/69	51/51		cefoxitin	100
mecA -	18/69		0/18		100
emrC +	34/69	33/34		erytromycin	97
emrC -	35/69		27/35		77
emrC +	34/69	33/34		clindamycin	97
emrC -	35/69		30/35		86
fosB +	22/69	22/22		fusidic acid	100
fosB -	47/69		44/47		94
aac(6')-aph(2'') +	58/69	58/58		gentamicin	100
aac(6')-aph(2'') -	11/69		8/11		73
Point mutation[a]					
gyrA[b] +	50/69	50/50		norfloxacin	100
gyrA[b] -	19/69		19/19		100
rpoB[c] +	23/69	23/23		rifampicin	100
rpoB[c] -	46/69		45/46		98
folA[d] +	40/69	40/69		trimethoprim-	100
folA[d] -	29/69		19/29[b]	sulfamethoxazole	66

[a]+ = presence; - = absence

[b]S84F (*n*=36); S84Y (*n*=14)

[c]D471E, I527M (*n*=17); H481N (*n*=4); S486F (*n*=1); S486Y (*n*=1)

[d]F99Y (*n*=40)
